# (*E*)-*N*′-Benzyl­idene-*p*-toluene­sulfono­hydrazide

**DOI:** 10.1107/S1600536808027219

**Published:** 2008-08-30

**Authors:** Hossein Mehrabi, Reza Kia, Ali Hassanzadeh, Samaneh Ghobadi, Hamid Reza Khavasi

**Affiliations:** a Department of Chemistry, Vali-e-Asr University of Rafsanjan, Rafsanjan, 77176, Iran; b X-ray Crystallography Unit, School of Physics, Universiti Sains Malaysia, 11800 USM, Penang, Malaysia; c Department of Chemistry, Faculty of Science, Urmia University, Urmia, Iran; d Department of Chemistry, Faculty of Science, Shahid Beheshti University, Tehran, Iran

## Abstract

In the title compound, C_14_H_14_N_2_O_2_S, a novel sulfonamide derivative, an intra­molecular C—H⋯O hydrogen bond generates an *S*(5) ring motif. The mol­ecule adopts a twisted *E* configuration around the C=N bond. An inter­molecular N—H⋯O hydrogen bond generates an *R* ^2^_2_(8) ring motif. The dihedral angle between the rings is 85.37 (9)°. The H atoms of the methyl group have rotational disorder with refined site occupancies of *ca* 0.63/0.37. In the crystal structure, inter­molecular N—H⋯O hydrogen bonds link neighbouring mol­ecules into dimers which stack along the *a *axis with a centroid–centroid distance of 3.8856 (10) Å.

## Related literature

For bond-length data, see: Allen *et al.* (1987 ▶). For hydrogen-bond motifs, see: Bernstein *et al.* (1995 ▶). For related structures and applications, see, for example: Tabatabaee *et al.* (2007 ▶); Ali *et al.* (2007 ▶); Tierney *et al.* (2006 ▶); Krygowski *et al.* (1998 ▶); Kayser *et al.* (2004 ▶).
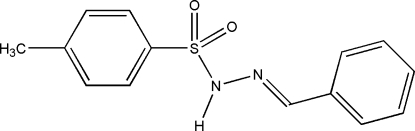


## Experimental

### 

#### Crystal data

C_14_H_14_N_2_O_2_S
*M* *_r_* = 274.33
Monoclinic, 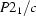

*a* = 5.9593 (7) Å
*b* = 9.6592 (7) Å
*c* = 23.712 (3) Å
β = 91.533 (9)°
*V* = 1364.4 (3) Å^3^
*Z* = 4
Mo *K*α radiation
μ = 0.24 mm^−1^
*T* = 293 (2) K
0.50 × 0.40 × 0.03 mm

#### Data collection

STOE IPDSII diffractometer
Absorption correction: numerical (*X-SHAPE*; Stoe & Cie, 2004 ▶) *T*_min_ = 0.879, *T*_max_ = 0.993
8939 measured reflections
3573 independent reflections
3008 reflections with *I* > 2σ(*I*)
*R*_int_ = 0.026

#### Refinement

*R*[*F* ^2^ > 2σ(*F* ^2^)] = 0.041
*wR*(*F* ^2^) = 0.110
*S* = 1.10
3573 reflections
195 parameters
H atoms treated by a mixture of independent and constrained refinement
Δρ_max_ = 0.23 e Å^−3^
Δρ_min_ = −0.25 e Å^−3^

### 

Data collection: *X-AREA* (Stoe & Cie, 2005 ▶); cell refinement: *X-AREA*; data reduction: *X-AREA*; program(s) used to solve structure: *SHELXTL* (Sheldrick, 2008 ▶); program(s) used to refine structure: *SHELXTL*; molecular graphics: *SHELXTL*; software used to prepare material for publication: *SHELXTL* and *PLATON* (Spek,2003 ▶).

## Supplementary Material

Crystal structure: contains datablocks global, I. DOI: 10.1107/S1600536808027219/at2617sup1.cif

Structure factors: contains datablocks I. DOI: 10.1107/S1600536808027219/at2617Isup2.hkl

Additional supplementary materials:  crystallographic information; 3D view; checkCIF report

## Figures and Tables

**Table 1:** Hydrogen-bond geometry (Å, °)

*D*—H⋯*A*	*D*—H	H⋯*A*	*D*⋯*A*	*D*—H⋯*A*
N1—H1*N*1⋯O1^i^	0.863 (19)	2.082 (19)	2.9446 (17)	177.0 (18)
C5—H5*A*⋯O2	0.93	2.54	2.9133 (18)	104

Symmetry code: (i) 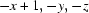.

**Fig. 1.**
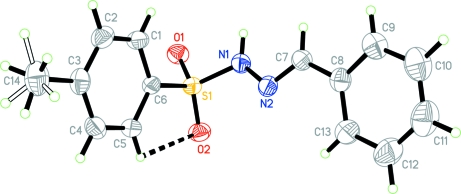
The molecular structure of the title compound, showing 40% probability displacement ellipsoids and the atomic numbering. Open bonds indicate the minor disordered component. Intramolecular hydrogen bond is shown as a dashed line.

**Fig. 2.**
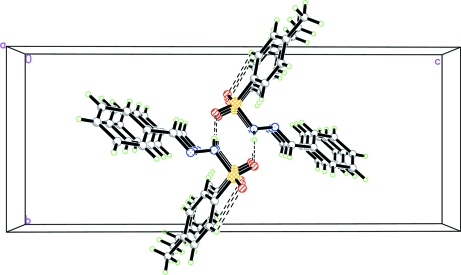
The crystal packing of the major component of (I), viewed down the a-axis, showing staking arrangement of molecules. Intermolecular and intermolecular hydrogen bonds are shown as dashed line.

## supplementary crystallographic information

### Comment

Sulfonamides were the first class of antimicrobial agents to be discovered. They inhibit dihydropteroate synthetase in the bacterial folic acid pathway. Although their clinical role has diminished, they are still useful in certain situations, because of its efficacy and low cost (Krygowski *et al.*, 1998). Sulfonamides (sulfanilamide, sulfamethoxazole, sulfafurazole) are structural analogs of *p*-aminobenzoic acid (PABA) and compete with PABA to block its conversion to dihydrofolic acid. These agents are generally used in combination with other drugs (usually sulfonamides) to prevent or treat a number of bacterial and parasitic infections (Tierney *et al.*, 2006). Some of the applications of sulfonamides are the anti-infective agents of choice, as follows: Bacteria as Human Pathogens, such as Antibiotic Treatment of Infections Caused by Gram-Positive Bacilli and Gram-negative Haemophilus ducreyi and Haemophilus aegyptius, Alternative Drug for treatment of Chlamydia related diseases (including C. trachomatis, Chlamydia psittaci, Chlamydia pneumonia), Anti-malarial Agents as Dihydropteroate synthetase inhibitors, alternative drugs in tuberculosis treatment, long term treatment of leprosy, treatment of ocular infections. In the latter treatment causative organisms must be identified, and it is preferable to use a drug that is not given systemically. Sulfonamides are also assumed as permitted antibiotics in Pregnancy (Kayser *et al.*, 2004).

The title compound (I) (Fig. 1), is a novel sulfonamide derivative. Bond lengths and angles are within the normal ranges (Allen *et al.*, 1987) and are comparable with the similar staructures (Ali *et al.* 2007). An intramolecular C—H···O hydrogen bond generate *S*(5) ring motif (Bernstein *et al.*, 1995). The molecule adopts a twisted *E* configuration around the C═N. An intermolecular N—H···O hydrogen bond genarate *R*^2^_2_(8) ring motif (Bernstein *et al.*, 1995). The dihedral angle between the phenyl rings is 85.37 (9)°. None of the H atoms of the methyl group was clearly resolved in the difference Fourier map and they were disordered over six positions and refined with site-occupancy factors of 0.62 (3) and 0.38 (3) for the major and minor componenets, respectively. In the crystal structure, intermolecular N—H···O interactions link neighbouring molecules into dimers which stacked along the *a*-axis. The short distance between the centroids of the six-membered rings prove an exsistence of the π-π interactions with centroid to centroid dsiatnce of 3.8856 (10) Å. The crystal structure is stabilized by intramolecular C—H···O, intermolecular N—H···O hydrogen bonds and π-π satcking.

### Experimental

*p*-Tosylhydrazine (3 mmol) was added to a stirred solution of benzaldehyde (3 mmol) in 2 ml of toluene at 20–25° C. The mixture was stirred for 6 h at 110° C. After cooling, the colorless crystalline solid was isolated by filtration, washed with cold toluene, and re-crystallized from ethanol.

### Refinement

H atom bound to N1 was located from a difference Fourier map and refined freely. The methyl hydrogen atoms were located from the difference Fourier map and refined freely with the parent atom. The rest of the hydrogen atoms were positioned geometrically and refined as riding model.

### Crystal data

**Table:**

C_14_H_14_N_2_O_2_S	*F*_000_ = 576
*M**_r_* = 274.33	*D*_x_ = 1.336 Mg m^−^^3^
Monoclinic, *P*2_1_/*c*	Mo *K*α radiation λ = 0.71073 Å
Hall symbol: -P 2ybc	Cell parameters from 2500 reflections
*a* = 5.9593 (7) Å	θ = 2.3–29.2º
*b* = 9.6592 (7) Å	µ = 0.24 mm^−^^1^
*c* = 23.712 (3) Å	*T* = 293 (2) K
β = 91.533 (9)º	Plate, colourless
*V* = 1364.4 (3) Å^3^	0.50 × 0.40 × 0.03 mm
*Z* = 4	

### Data collection

**Table:**

STOE IPDSII diffractometer	3573 independent reflections
Radiation source: fine-focus sealed tube	3008 reflections with *I* > 2σ(*I*)
Monochromator: graphite	*R*_int_ = 0.026
Detector resolution: 0.15 pixels mm^-1^	θ_max_ = 29.0º
*T* = 293(2) K	θ_min_ = 2.3º
φ scans	*h* = −8→6
Absorption correction: numerical(X-SHAPE; Stoe & Cie, 2004)	*k* = −12→13
*T*_min_ = 0.879, *T*_max_ = 0.993	*l* = −32→32
8939 measured reflections	

### Refinement

**Table:**

Refinement on *F*^2^	Secondary atom site location: difference Fourier map
Least-squares matrix: full	Hydrogen site location: inferred from neighbouring sites
*R*[*F*^2^ > 2σ(*F*^2^)] = 0.041	H atoms treated by a mixture of independent and constrained refinement
*wR*(*F*^2^) = 0.110	*w* = 1/[σ^2^(*F*_o_^2^) + (0.0507*P*)^2^ + 0.2385*P*] where *P* = (*F*_o_^2^ + 2*F*_c_^2^)/3
*S* = 1.10	(Δ/σ)_max_ < 0.001
3573 reflections	Δρ_max_ = 0.23 e Å^−^^3^
195 parameters	Δρ_min_ = −0.25 e Å^−^^3^
Primary atom site location: structure-invariant direct methods	Extinction correction: none

### Special details

**Table:**

Geometry. All e.s.d.'s (except the e.s.d. in the dihedral angle between two l.s. planes) are estimated using the full covariance matrix. The cell e.s.d.'s are taken into account individually in the estimation of e.s.d.'s in distances, angles and torsion angles; correlations between e.s.d.'s in cell parameters are only used when they are defined by crystal symmetry. An approximate (isotropic) treatment of cell e.s.d.'s is used for estimating e.s.d.'s involving l.s. planes.
Refinement. Refinement of *F*^2^ against ALL reflections. The weighted *R*-factor *wR* and goodness of fit *S* are based on *F*^2^, conventional *R*-factors *R* are based on *F*, with *F* set to zero for negative *F*^2^. The threshold expression of *F*^2^ > σ(*F*^2^) is used only for calculating *R*-factors(gt) *etc*. and is not relevant to the choice of reflections for refinement. *R*-factors based on *F*^2^ are statistically about twice as large as those based on *F*, and *R*- factors based on ALL data will be even larger.

### Fractional atomic coordinates and isotropic or equivalent isotropic displacement parameters (Å^2^)

**Table:**

	*x*	*y*	*z*	*U*_iso_*/*U*_eq_	Occ. (<1)
S1	0.24869 (6)	0.18884 (4)	0.001194 (15)	0.04475 (12)	
O1	0.39754 (19)	0.13796 (12)	−0.04087 (4)	0.0555 (3)	
O2	0.03368 (18)	0.24148 (12)	−0.01555 (5)	0.0572 (3)	
N1	0.2191 (2)	0.05429 (14)	0.04190 (6)	0.0531 (3)	
H1N1	0.328 (3)	−0.004 (2)	0.0425 (8)	0.063 (5)*	
N2	0.0856 (2)	0.06535 (13)	0.08855 (6)	0.0500 (3)	
C1	0.6026 (2)	0.28675 (16)	0.06340 (7)	0.0504 (3)	
H1A	0.6706	0.2023	0.0558	0.060*	
C2	0.7124 (3)	0.38438 (18)	0.09592 (7)	0.0565 (4)	
H2A	0.8548	0.3648	0.1108	0.068*	
C3	0.6147 (3)	0.51220 (17)	0.10705 (6)	0.0542 (4)	
C4	0.4028 (3)	0.53993 (16)	0.08404 (7)	0.0551 (4)	
H4A	0.3367	0.6255	0.0905	0.066*	
C5	0.2883 (2)	0.44255 (15)	0.05169 (6)	0.0480 (3)	
H5A	0.1460	0.4619	0.0367	0.058*	
C6	0.3884 (2)	0.31589 (14)	0.04191 (6)	0.0418 (3)	
C7	0.1133 (3)	−0.03135 (15)	0.12464 (7)	0.0509 (3)	
H7A	0.2260	−0.0962	0.1192	0.061*	
C8	−0.0264 (3)	−0.04390 (16)	0.17434 (6)	0.0516 (3)	
C9	0.0359 (3)	−0.1379 (2)	0.21640 (7)	0.0655 (4)	
H9A	0.1678	−0.1885	0.2134	0.079*	
C10	−0.0980 (5)	−0.1566 (3)	0.26284 (8)	0.0807 (6)	
H10A	−0.0555	−0.2196	0.2908	0.097*	
C11	−0.2921 (5)	−0.0825 (3)	0.26759 (9)	0.0884 (7)	
H11A	−0.3815	−0.0953	0.2987	0.106*	
C12	−0.3558 (4)	0.0115 (3)	0.22621 (10)	0.0858 (6)	
H12A	−0.4876	0.0620	0.2296	0.103*	
C13	−0.2239 (3)	0.0303 (2)	0.17986 (8)	0.0677 (5)	
H13A	−0.2679	0.0933	0.1521	0.081*	
C14	0.7396 (5)	0.6169 (3)	0.14314 (10)	0.0776 (6)	
H14A	0.651 (10)	0.695 (7)	0.147 (3)	0.116*	0.63 (4)
H14B	0.855 (10)	0.656 (7)	0.124 (2)	0.116*	0.63 (4)
H14C	0.789 (11)	0.581 (6)	0.177 (2)	0.116*	0.63 (4)
H14D	0.73 (2)	0.714 (12)	0.128 (4)	0.116*	0.37 (4)
H14E	0.920 (16)	0.579 (9)	0.151 (4)	0.116*	0.37 (4)
H14F	0.648 (17)	0.625 (10)	0.185 (4)	0.116*	0.37 (4)

### Atomic displacement parameters (Å^2^)

**Table:**

	*U*^11^	*U*^22^	*U*^33^	*U*^12^	*U*^13^	*U*^23^
S1	0.04108 (18)	0.04409 (19)	0.04919 (19)	0.00320 (13)	0.00301 (13)	−0.00108 (14)
O1	0.0593 (6)	0.0565 (6)	0.0512 (5)	0.0086 (5)	0.0097 (5)	−0.0032 (5)
O2	0.0440 (6)	0.0602 (6)	0.0668 (7)	0.0048 (5)	−0.0070 (5)	−0.0030 (5)
N1	0.0518 (7)	0.0427 (6)	0.0656 (8)	0.0037 (5)	0.0150 (6)	0.0018 (6)
N2	0.0461 (6)	0.0451 (6)	0.0592 (7)	−0.0026 (5)	0.0096 (5)	−0.0020 (5)
C1	0.0421 (7)	0.0527 (8)	0.0564 (8)	0.0078 (6)	0.0017 (6)	0.0020 (6)
C2	0.0445 (7)	0.0672 (10)	0.0573 (8)	−0.0030 (7)	−0.0046 (6)	0.0058 (7)
C3	0.0606 (9)	0.0558 (8)	0.0463 (7)	−0.0140 (7)	0.0025 (6)	0.0044 (6)
C4	0.0635 (9)	0.0421 (7)	0.0600 (8)	0.0010 (6)	0.0065 (7)	0.0005 (6)
C5	0.0424 (7)	0.0448 (7)	0.0568 (8)	0.0051 (5)	0.0011 (6)	0.0043 (6)
C6	0.0376 (6)	0.0437 (7)	0.0442 (6)	0.0017 (5)	0.0049 (5)	0.0045 (5)
C7	0.0494 (8)	0.0433 (7)	0.0601 (8)	−0.0005 (6)	0.0041 (6)	−0.0039 (6)
C8	0.0559 (8)	0.0472 (7)	0.0515 (7)	−0.0083 (6)	0.0007 (6)	−0.0062 (6)
C9	0.0745 (11)	0.0648 (10)	0.0568 (9)	−0.0035 (9)	−0.0053 (8)	−0.0001 (8)
C10	0.1072 (17)	0.0847 (14)	0.0501 (9)	−0.0107 (12)	−0.0013 (10)	0.0050 (9)
C11	0.1082 (18)	0.1013 (17)	0.0570 (10)	−0.0159 (14)	0.0253 (11)	−0.0081 (11)
C12	0.0802 (14)	0.0963 (16)	0.0823 (13)	0.0054 (12)	0.0281 (11)	−0.0057 (12)
C13	0.0655 (11)	0.0687 (11)	0.0695 (10)	0.0047 (8)	0.0135 (8)	0.0040 (9)
C14	0.0961 (17)	0.0716 (13)	0.0646 (11)	−0.0275 (12)	−0.0085 (11)	−0.0039 (10)

### Geometric parameters (Å, °)

**Table:**

S1—O2	1.4250 (11)	C7—H7A	0.9300
S1—O1	1.4389 (11)	C8—C13	1.387 (3)
S1—N1	1.6313 (14)	C8—C9	1.392 (2)
S1—C6	1.7573 (14)	C9—C10	1.389 (3)
N1—N2	1.3840 (18)	C9—H9A	0.9300
N1—H1N1	0.86 (2)	C10—C11	1.368 (4)
N2—C7	1.274 (2)	C10—H10A	0.9300
C1—C2	1.373 (2)	C11—C12	1.382 (3)
C1—C6	1.3908 (19)	C11—H11A	0.9300
C1—H1A	0.9300	C12—C13	1.380 (3)
C2—C3	1.393 (2)	C12—H12A	0.9300
C2—H2A	0.9300	C13—H13A	0.9300
C3—C4	1.388 (2)	C14—H14A	0.93 (7)
C3—C14	1.509 (2)	C14—H14B	0.92 (6)
C4—C5	1.382 (2)	C14—H14C	0.92 (6)
C4—H4A	0.9300	C14—H14D	1.00 (11)
C5—C6	1.3832 (19)	C14—H14E	1.15 (10)
C5—H5A	0.9300	C14—H14F	1.15 (10)
C7—C8	1.466 (2)		
			
O2—S1—O1	119.68 (7)	C10—C9—H9A	119.8
O2—S1—N1	109.79 (7)	C8—C9—H9A	119.8
O1—S1—N1	102.50 (7)	C11—C10—C9	120.2 (2)
O2—S1—C6	108.24 (7)	C11—C10—H10A	119.9
O1—S1—C6	109.13 (7)	C9—C10—H10A	119.9
N1—S1—C6	106.76 (7)	C10—C11—C12	120.1 (2)
N2—N1—S1	119.01 (10)	C10—C11—H11A	119.9
N2—N1—H1N1	119.1 (13)	C12—C11—H11A	119.9
S1—N1—H1N1	116.1 (13)	C13—C12—C11	120.0 (2)
C7—N2—N1	114.41 (13)	C13—C12—H12A	120.0
C2—C1—C6	119.05 (14)	C11—C12—H12A	120.0
C2—C1—H1A	120.5	C12—C13—C8	120.64 (19)
C6—C1—H1A	120.5	C12—C13—H13A	119.7
C1—C2—C3	121.33 (14)	C8—C13—H13A	119.7
C1—C2—H2A	119.3	C3—C14—H14A	109 (4)
C3—C2—H2A	119.3	C3—C14—H14B	111 (3)
C4—C3—C2	118.47 (14)	H14A—C14—H14B	99 (4)
C4—C3—C14	121.57 (19)	C3—C14—H14C	113 (3)
C2—C3—C14	119.96 (19)	H14A—C14—H14C	112 (5)
C5—C4—C3	121.13 (15)	H14B—C14—H14C	112 (5)
C5—C4—H4A	119.4	C3—C14—H14D	113 (5)
C3—C4—H4A	119.4	H14A—C14—H14D	41 (6)
C4—C5—C6	119.10 (14)	H14B—C14—H14D	59 (5)
C4—C5—H5A	120.4	H14C—C14—H14D	133 (6)
C6—C5—H5A	120.4	C3—C14—H14E	109 (4)
C5—C6—C1	120.88 (14)	H14A—C14—H14E	141 (5)
C5—C6—S1	120.65 (11)	H14B—C14—H14E	60 (4)
C1—C6—S1	118.47 (11)	H14C—C14—H14E	58 (4)
N2—C7—C8	122.31 (14)	H14D—C14—H14E	114 (7)
N2—C7—H7A	118.8	C3—C14—H14F	108 (4)
C8—C7—H7A	118.8	H14A—C14—H14F	64 (5)
C13—C8—C9	118.70 (16)	H14B—C14—H14F	141 (5)
C13—C8—C7	122.31 (15)	H14C—C14—H14F	54 (4)
C9—C8—C7	118.94 (16)	H14D—C14—H14F	102 (7)
C10—C9—C8	120.3 (2)	H14E—C14—H14F	110 (6)
			
O2—S1—N1—N2	−52.51 (14)	N1—S1—C6—C5	−119.42 (12)
O1—S1—N1—N2	179.27 (11)	O2—S1—C6—C1	179.55 (11)
C6—S1—N1—N2	64.61 (13)	O1—S1—C6—C1	−48.69 (13)
S1—N1—N2—C7	−164.31 (11)	N1—S1—C6—C1	61.41 (13)
C6—C1—C2—C3	−0.9 (2)	N1—N2—C7—C8	−174.66 (13)
C1—C2—C3—C4	−0.5 (2)	N2—C7—C8—C13	13.1 (2)
C1—C2—C3—C14	179.77 (17)	N2—C7—C8—C9	−169.75 (15)
C2—C3—C4—C5	1.2 (2)	C13—C8—C9—C10	0.0 (3)
C14—C3—C4—C5	−179.06 (17)	C7—C8—C9—C10	−177.21 (16)
C3—C4—C5—C6	−0.5 (2)	C8—C9—C10—C11	−0.1 (3)
C4—C5—C6—C1	−1.0 (2)	C9—C10—C11—C12	−0.1 (4)
C4—C5—C6—S1	179.82 (11)	C10—C11—C12—C13	0.3 (4)
C2—C1—C6—C5	1.7 (2)	C11—C12—C13—C8	−0.3 (3)
C2—C1—C6—S1	−179.11 (12)	C9—C8—C13—C12	0.1 (3)
O2—S1—C6—C5	−1.28 (14)	C7—C8—C13—C12	177.28 (18)
O1—S1—C6—C5	130.48 (12)		

### Hydrogen-bond geometry (Å, °)

**Table:**

*D*—H···*A*	*D*—H	H···*A*	*D*···*A*	*D*—H···*A*
N1—H1N1···O1^i^	0.863 (19)	2.082 (19)	2.9446 (17)	177.0 (18)
C5—H5A···O2	0.93	2.54	2.9133 (18)	104

Symmetry codes: (i) −*x*+1, −*y*, −*z*.
